# Sodium-Directed Crosstalk Between Immune Cells and Lymphatic Vessels

**DOI:** 10.1007/s11906-024-01322-3

**Published:** 2025-01-15

**Authors:** Taseer Ahmad, Rachelle Crescenzi, Valentina Kon, Annet Kirabo, Elaine L. Shelton

**Affiliations:** 1 Department of Pharmacology, College of Pharmacy, University of Sargodha, Sargodha, 40100 Pakistan; 2 Division of Clinical Pharmacology, Department of Medicine, Vanderbilt University Medical Center, Nashville, TN USA; 3 Department of Radiology and Radiological Sciences, Vanderbilt University Medical Center, Nashville, TN USA; 4 Department of Biomedical Engineering, Vanderbilt University, Nashville, TN USA; 5 Department of Pediatrics, Vanderbilt University Medical Center, Nashville, TN USA; 6 Department of Molecular Physiology and Biophysics, Vanderbilt University, Nashville, TN 37232 USA; 7 Vanderbilt Center for Immunobiology, Nashville, USA; 8 Vanderbilt Institute for Infection, Immunology and Inflammation, Nashville, USA; 9 Vanderbilt Institute for Global Health, Nashville, USA

**Keywords:** Sodium, Immune Cells, Lymphatic vessels, Kidney diseases, Salt-sensitive blood pressure

## Abstract

**Purpose of Review:**

The role of the lymphatic system in clearing extravasated fluids, lipid transport, and immune surveillance is well established, and lymphatic vasculature can provide a vital role in facilitating crosstalk among various organ systems. Lymphatic vessels rely on intrinsic and local factors to absorb and propel lymph from the interstitium back to the systemic circulation. The biological implications of local influences on lymphatic vessels are underscored by the exquisite sensitivity of these vessels to environmental stimuli. This review is intended to highlight the role of sodium within the local environment in mediating lymphatic and immune cell interactions that contribute to changes in function and disease progression.

**Recent Findings:**

We discuss evidence that accumulation of interstitial sodium modulates lymphatic growth, pumping dynamics, and permeability of renal lymphatics, which involves activation of sodium potassium chloride co-transporter (NKCC1) in lymphatic endothelial cells. These recent findings complement observations that sodium activates immune cells via the epithelial sodium channel (ENaC), leading to the formation and accumulation of lipid oxidation products, isolevuglandins (IsoLGs), in antigen presenting cells, which in turn promotes T cell activation and vasculopathy. In addition, we will underscore the physiologic relevance of altered interplay between immune cells and lymphatics in the sodium avid state that characterizes kidney diseases and consider how sodium accumulation in the interstitial compartment of the kidney modulates the lymphatic network and the interactions between renal lymphatics and activated immune cells.

**Summary:**

Finally, this article calls attention to persisting knowledge gaps and stresses the need for additional studies to identify salt-sensing mechanisms, including sodium-activated immune cells and lymphatic endothelial cell interactions, for targeted therapeutic interventions in the setting of renal disease.

## Introduction

The lymphatic system plays a critical role in preserving fluid balance, transporting lipids, and maintaining immune surveillance. Interstitial fluid containing macromolecules, solutes, and immune cells (collectively referred to as lymph) enters the lymphatics via blind-ended capillaries, which course through virtually every tissue in the body [1]. Lymphatic capillaries are composed of loose overlapping endothelial cells with button-like cell junctions and anchoring filaments tethered to the interstitial extracellular matrix. When interstitial fluid accumulates, the matrix swells, causing the filaments to tug on lymphatic endothelial cells (LECs), creating spaces between cells, which increases permeability and prevents capillary collapse [2]. Lymph drains from the capillary plexus into larger pre-collecting and collecting vessels, which transport lymph over long distances. The structure of these vessels reflects their function in that they are lined with relatively impermeable LECs containing continuous zipper-like cell junctions, wrapped in contractile lymphatic smooth muscle cells and studded with intraluminal valves, which coordinate to propel lymph towards the thoracic duct, where it re-enters the systemic circulation at the junction of the left subclavian and internal jugular veins [3].

In addition to regulating interstitial fluid balance, the lymphatic system plays an active role in lipid absorption and transport. Dietary fat is absorbed from the lumen of the small intestine into enterocytes where it is packaged into lipoprotein particles know as chylomicrons, composed of triglycerides, phospholipids, cholesterol, and proteins. Chylomicrons then enter the lymphatic system through intestinal lacteals, where they are carried in the lymph through collecting vessels and lymph nodes until they enter the blood, which facilitates their dispersion throughout the body. Additionally, the lymphatic system regulates the removal of excess lipids from peripheral tissues, a process known as reverse cholesterol transport. In the interstitium, excess cholesterol is incorporated into high-density lipoprotein particles that drain into lymphatic capillary beds and are transported back to the systemic circulation, where they enter the liver and are converted into bile salts for excretion [4–6].

Another critical function of the lymphatic system comes via its role in immune surveillance and adaptive immunity. Lymph provides important information about the tissue from which it came and is compositionally and functionally distinct from blood plasma, with enrichment in humoral factors, antigens, and antigen presenting cells (APCs). A proteomic analysis comparing matched lymph and plasma samples revealed lymph was enriched in proteins involved in apoptosis, cell metabolism/catabolism, and extracellular matrix remodeling [7]. Moreover, lymph contains tissue-specific proteins that map to the organs from which the lymph originated, suggesting that lymph is uniquely defined by the anatomical region from which it was derived [8]. Importantly, studies have demonstrated that the protein content of lymph can be modulated by pathologic conditions including sepsis, pancreatitis, asthma, and trauma/hemorrhage. Under these conditions, lymph becomes enriched in proteins related to cell lysis products, microbial components like LPS, mediators of inflammation, and energy/redox metabolism [9–12]. Lymph also contains tissue-resident immune cells, primarily dendritic cells (DCs) and memory T-cells, that are transported from the periphery to lymph nodes via collecting vessels. As lymph enters the node, free antigens and activated APCs are concentrated and introduced to naïve T-cells, while larger antigens and microbial particles are sampled by macrophages and B-cells [13]. The collective interplay between the lymphatic vasculature, lymph, and lymph nodes provides a highly organized structure for fine-tuning immune responses to foreign agents while preventing inappropriate reactions to self-antigens.

Proper lymphatic function is dependent on regulatory cues from the local environment. Lymphatic vessels are uniquely sensitive to their microenvironment, adjusting rapidly to even slight alterations in interstitial pressure (transmural and intraluminal pressure), surrounding tissue temperature, lymph flow rates (fluid shear stress), and lymph composition (osmolarity), by changing permeability and pumping efficiency parameters [14]. This allows lymphatic vessels to proactively adapt to the needs of the local tissue state (fluid and solute removal) in real time. The lymphatic system plays a unique and integral role in facilitating interorgan crosstalk, providing the primary link between the body’s peripheral organ systems, immune system, and the blood circulation. Thus, pathologic changes in an organ’s microenvironment or local lymph composition that cause downstream disruptions in lymphatic permeability, pumping dynamics, or immune cell interactions could have widespread consequences for other organ systems. This review illustrates this concept by highlighting the effects of sodium on the relationship between lymphatics and immune cells in the setting of kidney disease.

### Kidney Disease is a Sodium Avid State

Sodium is fundamental to osmoregulation and transmembrane ion gradients essential in cellular functions. Accumulation of sodium is both a driver and consequence of disease progression in hypertension, congestive heart failure, and chronic kidney disease [15, 16]. Classically, sodium accumulation is accompanied by retention of water that is clinically manifested as edema [17]. However, increased sodium levels in the circulating plasma do not reflect sodium accumulation. Indeed, plasma sodium concentration remains remarkably stable across various disease conditions and fluctuating salt intake. Instead, total body sodium accumulation is thought to reflect sodium accrual in the interstitial compartment, which has been documented to reach as high as 250 mM [14, 18]. Studies have shown interstitial sodium storage in the skin and muscle is electrostatically associated with glycosaminoglycans. Machnik et al. reported that excess dietary salt in rodents increases interstitial sodium in the skin without changing plasma concentration, reiterating the extravascular nature of sodium accumulation [19, 20]. Subsequent studies using ^23^Na magnetic resonance imaging (MRI) found that sodium accumulates in the skin and skeletal muscle of humans with hypertension, chronic kidney disease, heart failure, and advanced age [21]. More recent studies have found that sodium accumulation may not be hypertonic and that the accumulation of salt, together with water, is not confined to skin and muscle but rather systemic, affecting other organs, including lung, liver, and heart [22, 23]. Using multi-nuclear ^23^Na/1H MRI at baseline and after puromycin- proteinuric injury in the rat (PAN), sodium and water were increased in the cortex compared to the papilla region in the injured versus control kidneys. This analysis method has several advantages for probing sodium content in the kidney over other methods [24] Sodium ^23^Na is the second most abundant endogenous magnetic nuclei after 1 H protons abundant in water. Also, sodium can be imaged noninvasively at relatively high spatial resolutions in vivo. The imaging findings of increased sodium were supported by data showing that lymph exiting the proteinuric kidneys had higher sodium concentration compared with renal lymph exiting the kidneys of normal uninjured animals. In contrast, sodium levels in the circulating plasma of proteinuric and normal uninjured animals were not different. Increased sodium content has been reported in the kidney lymph in another example of sodium avid state, the canine experimental model of heart failure [25, 26]. Moreover, the findings in renal lymph are similar to results in lymph collected from dermal lymphatic vessels of salt sensitive hypertensive rats which showed increased lymphatic sodium concentration but no change in the plasma sodium levels [27]. Cumulatively, these observations illustrate an essential concept - that despite relatively constant sodium levels in the circulation, specific interstitial compartments may contain different sodium concentrations.

**Fig. 1:**
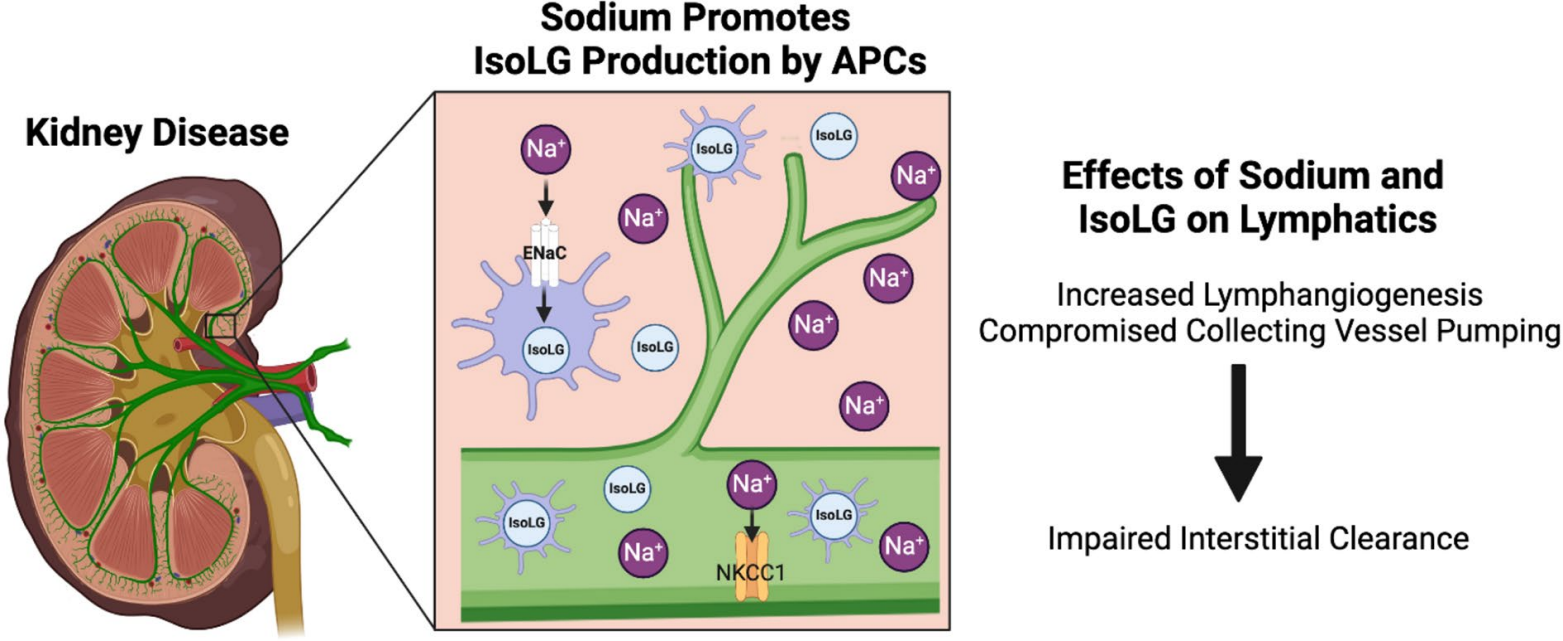
Kidney disease-driven sodium accumulation modulates lymphatic endothelial and infiltrating immune cell function and crosstalk. Kidney disease promotes renal accumulation of sodium and IsoLG. Sodium enters antigen-presenting cells (APCs) through the epithelial sodium channel (ENaC), leading to the production of IsoLG. Increased sodium and IsoLG impair renal lymphatic vessel function through pathways involving NKCC1 signaling. Crosstalk between these distinct systems within the diseased kidney alters the local environment and can modulate disease progression

The notion of the regulatory role of the local environment on lymphatic function has important biological ramifications, particularly in the context of sodium avid states. Unlike blood vessels, which depend on a central pump (heart) for blood circulation, the lymphatic vessels rely on intrinsic factors such as vascular contractility and integrity of lymphatic valves together with extrinsic factors, such as interstitial pressure, shear stress, temperature, osmolarity, and sodium concentration to propel lymph flow from the interstitium to the systemic circulation. The intrinsic and extrinsic mechanisms usually synchronize, but their relative importance varies among different tissues and different physiologic/pathophysiologic conditions. Nonetheless, this regulatory framework allows the ambient microenvironment to tune the uptake and propulsion of lymph (potentially by local osmotic gradients between interstitium and lymphatic capillaries) and subsequently, clearance of the interstitium. The established view that kidney disease drives accumulation of total body sodium appears to include sodium enrichment within the kidneys themselves. Given that lymphatic vessels are exquisitely sensitive to local environmental stimuli [13], the increased sodium within the kidney microenvironment can therefore influence lymphatic architecture and functionality observed locally as well as in distant tissues. Our group has previously established sodium as a critical factor in immune cell activation that underlies hypertension-induced vasculopathy [24]. This important observation raises the possibility that aside from influencing renal lymphatics directly, intrarenal sodium also modulates the interactions between the lymphatics and activated immune cells infiltrating injured kidneys which can have more widespread ramifications, see Fig. 1.

## Sodium Regulates Lymphatic Vessel Growth and Dynamics

Sodium regulates the growth of the lymphatic vascular network. This effect has been most extensively studied in the skin of hypertensive animals and humans and involves transcription factor tonicity-responsive enhancer protein (TonEBP)-induced macrophage secretion of vascular endothelial growth factor-C (VEGF-C) [27]. Macrophage depletion or blockade of VEGF-C signaling diminishes lymphangiogenesis [28]. It appears that, as in other tissues, sodium stimulation of immune cells to produce VEGF-C contributes to renal lymphangiogenesis. Numerous experimental kidney injury models and human kidney diseases have documented renal lymphangiogenesis [24, 29–32]. In unilateral ureteral obstruction (UUO) models, VEGF-C released from macrophages drives lymphangiogenesis which reflects macrophage produced VEGF-C activation of CCR2-mediated PI3K-AKT-mTOR signaling pathway as well as through hyaluronan and toll-like receptor 4-dependent signaling [30, 33–35]. T-cells also modulate lymphatic growth. Interestingly, while regulatory T cells increase lymphatic growth through lymphotoxin β receptor signaling in LECs, Th2 cells produce cytokines that inhibit lymphangiogenesis [36]. Aside from immune cells and relevant to this discussion, VEGF-C as well as another lymphangiogenic factor, VEGF-D, are also produced by proximal tubule epithelial cells. Interestingly, kidney-specific overexpression of VEGF-D or targeted delivery of VEGF-C nanoparticles was shown to expand the kidney lymphatic network. This approach has proven effective in promoting sodium excretion and lowering systemic blood pressure in several hypertensive mouse models [37–39]. Importantly, elevated levels of systemic VEGF-C and -D may stimulate lymphangiogenesis outside of the kidney. Indeed, proteinuric rats and mice were found to have intestinal lymphangiogenesis together with other changes in mesenteric lymphatics [40, 41]. It is, therefore, possible that sodium avid states, including kidney diseases, promote the expansion of the lymphatic vascular network in distant tissues and organs, which may represent a novel pathway for interorgan crosstalk.

Although kidney disease and, specifically increased interstitial sodium, is well accepted as causing growth of the lymphatic vascular network, less is understood about whether kidney disease or sodium-induced lymphangiogenesis is accompanied by disruption in lymphatic dynamics or resorptive capacity. A few studies have documented that a high salt diet can modulate lymphatic contractility [24, 42–44]. For example, fluorescent imaging of skin lymphatic vessels in high salt-fed mice and rats showed a dilation of lymphatic vessels coupled with a compensatory increase in lymphatic contraction frequency [42]. Isolated afferent iliac lymphatics from high salt-fed mice had blunted amplitude, ejection fraction, and stroke volume, leading to marked reductions in pumping efficiency [43]. Furthermore, animals fed a high salt diet, or deoxycorticosterone acetate (DOCA) treatment that increased sodium in skin and muscle, showed an overall increase in lymph flow [44]. However, this study also showed that direct exposure of mouse inguinal afferent lymphatic vessels to high sodium increased contraction frequency but reduced the magnitude of contraction such that only weak contractions were observed [44]. Furthermore, renal collecting lymphatic vessels directly exposed to a high sodium environment had little change in contraction frequency but a pronounced increase in end-systolic diameter (ESD) that contributed to reduced contraction amplitude and ejection fraction [24]. Although more studies are needed, the existing literature suggests salt avid states can lead to increased lymph flow, possibly reflecting a response to the expanded extracellular compartment. However, under certain circumstances increased sodium can cause specific disruptions in lymphatic contractile dynamics that blunt efficient clearance of the interstitium. Together, these results support the concept that although sodium and injury reduce ejection fraction which is expected to impair clearance of the interstitial compartment, these involve different mechanisms which may become additive in the kidney disease setting.

Molecularly, lymphatic contractility is regulated by action potentials that trigger Ca^++^ influx generated by ion channels and transporters. The Na^+^-K^+^-2Cl^−^ cotransporters NKCC1 and NKCC2 play essential roles in regulating ion flux in a variety of cell types. NKCC2 is best known for its actions on the thick ascending limb of the loop of Henle where it regulates sodium homeostasis. Diuretics, including furosemide and bumetanide, inhibit both NKCC isoforms and are commonly used clinically to increase renal excretion of water and solutes from the body. NKCC1 has varied biological functions which include regulation of vascular tone [24, 45]. Lymphatic vessels express NKCC1 but not NKCC2 [24]. Studies on cultured LECs indicate that a high sodium environment can reduce NKCC1 activity by down regulating Ste20-related proline-alanine-rich kinase (SPAK), an upstream activating kinase of NKCC1. In lymphatic collecting vessels, NKCC1-inhibition via furosemide caused blunted pumping dynamics [46]. Notably, inhibition of NKCC1 with furosemide had a weaker effect on the pumping dynamics in renal collecting lymphatic vessels from kidney-injured animals than on vessels of uninjured control animals [24]. This is important because inhibition of NKCC cotransporters with furosemide is the first-line therapy to reduce sodium overload and fluid accumulation in a variety of diseases, including kidney disease. Thus, impaired lymphatic pumping, together with a high sodium environment characterizing kidney diseases, may diminish the therapeutic effects of NKCC inhibition. This very scenario may contribute to the relative resistance to diuretic therapy observed in patients with chronic kidney disease. Currently, therapeutic resistance centers on impaired delivery of the therapeutic agent to the relevant tubular segment. It is possible that the dysfunction of renal lymphatic vessels is related to electrolyte abnormalities in the microenvironment of the kidney, which could be a novel target to optimize removal of excess tissue sodium.

### Sodium Regulation of Immune Cells and Impact on Disease Progression

Increased sodium promotes inflammation by modulating the differentiation, activation, and function of various innate and adaptive immune cell types [47]. The effects of sodium on macrophage phenotype was recently described in an elegant study of salt-sensitive hypertensive mice, where renal and reproductive dysfunction coincided with preferential production of M1 macrophages via p38/MAPK-dependent NFAT5 transcriptional activation, increased inflammation, and elevated lymphangiogenesis in the gonads and kidney [48]. This phenotype could be therapeutically rescued using an angiotensin-(1–7) mimetic and Mas receptor agonist that promoted a shift from proinflammatory M1 to anti-inflammatory M2 macrophages [49]. Treated mice had reduced inflammation, reduced lymphangiogenesis, and improved renal and reproductive function, suggesting macrophage polarization could be a promising therapeutic target for improving sodium and hypertension-related organ damage.

Sodium has similar effects on inducing pro-inflammatory phenotypes in T cell subtypes including Th17, Th2, and Treg cells, although this process is fine-tuned by cytokine contributions as well. For example, in the presence of TGF-b or IL-6, sodium increases production of pro-inflammatory pathologic Th17 cells via activation of NKCC1, p38/MAPK, NFAT5, SGK1 pathways, while in the absence of polarizing cytokines, sodium instead activates FoxP3 and anti-inflammatory Th17 cells predominate [50].

In contrast to macrophages and T cells, sodium inhibits p38/MAPK and NFAT5 pathways in B cells [51]. In cell culture studies, high sodium exposure decreased B cell survival and differentiation. Interestingly, in mouse models of lupus featuring increased renal sodium concentration and nephritis, B cell survival and infiltration into the kidney is enhanced via activation of the sodium-potassium pump, Na^+^-K^+^-ATPase. Inhibiting this pump decreased the number of intrarenal B cells and reduced the extent of proteinuria in the animals [52].

Another mechanism by which sodium can modulate immune cells is by increasing production of reactive oxygen species (ROS), leading to isolevuglandin (IsoLG) formation in immune cells. IsoLGs are generated by peroxidation of arachidonic acid, resulting in highly reactive dicarbonyls that form covalent bonds with lysine residues leading to biologically relevant post-translational protein modifications [53]. In dendritic cells (DCs), sodium enters via ENaC channels and stimulates NADPH oxidase-mediated production of ROS. This in turn, causes IsoLG to accumulate, leading to production of IL-6, IL-1β, and IL-23, and subsequent activation of pro-inflammatory T cells [54]. In mice, adoptive transfer of salt-exposed DCs primes hypertension in response to a sub-pressor dose of angiotensin II [54]. Moreover, IsoLG-protein adduct formation is absent in mice lacking NADPH oxidase, and pharmacological scavenging of IsoLGs prevents DC activation, hypertension, and end-organ damage [54, 55].

IsoLGs are elevated in atherosclerosis, cardiac arrythmias, hypertension, cancer, pulmonary and liver fibrosis and IsoLG inhibitors have been shown to benefit several of these disease entities [56]. Importantly, studies have demonstrated that kidney disease not only promotes renal accumulation of sodium, but also the accumulation of renal IsoLG [24](. Both proteinuric animal models and humans with proteinuric kidney disease have increased urinary IsoLGs associated with ApoAI, the major protein in high density lipoprotein, known to be especially susceptible to modification by IsoLG. In fact, the IsoLG in urine of proteinuric humans appeared preferentially adducted to apoAI and not to other urinary proteins [57]. IsoLG-apoAI as well as the unmodified apoAI are taken up by proximal tubular epithelial cells and transported by renal lymphatics for return into the circulation. But the IsoLG-modified apoAI is more avidly taken up by the proximal tubules. Once within the interstitium, IsoLG can directly blunt lymphatic contractility and impair renal clearance. Indeed, the renal clearance of IsoLG-apoAI is slower than unmodified apoAI [58].

Collectively, it is clear that sodium is vital in regulating immunometabolism, and increased sodium concentrations can shift the immune cell balance toward inflammation, leading to significant effects on disease progression [59]. Elevated sodium can also influence the proliferation and differentiation of lymphatic endothelial cells, regulate lymphangiogenesis, and influence lymphatic vessel pumping dynamics [24, 59, 60]. Therefore, it is imperative to consider how immune cells and lymphatic vessels interact under normal and pathologic conditions in order to fully understand the impact of sodium on disease progression.

## Consequences of Immune Cell: Lymphatic Crosstalk

Beyond simply transporting cells, accumulating evidence indicates that lymphatic vessels affect the survival, activation, and proliferation of immune cells. Conversely, immune cells impact LEC proliferation and have profound effects on lymphatic vessel integrity. Both the immune cells and lymphatics are modulated by a high sodium environment. As a sodium avid state, kidney disease induces a high sodium environment within the injured kidneys that can in turn influence infiltrating immune cells, the renal lymphatics, and the crosstalk between these systems (Fig. 1**)**. As discussed above, in the context of kidney disease, both immune cells and tubular epithelial cells synthesize VEGF-C and VEGF-D which promote expansion and remodeling of the renal lymphatic capillaries through activation of VEGFR-3 [61, 62]. While kidney disease stimulation of kidney lymphangiogenesis is indisputable and linked to increased levels of VEGF-C and -D, the ramifications of the renal lymphangiogenic response are still unresolved. A protective effect has been suggested based on studies in mice with UUO injury treated with recombinant human VEGF-C, which reduced levels of pro-fibrotic TGFβ interstitial fibrosis thought to clear inflammatory cells and profibrotic molecules from the kidney interstitium [63]. Another study reported that podocyte-specific overexpression of VEGF-C in mice led to a reduction in glomerular injury and albuminuria [64]. On the other hand, functional impairment of newly formed or existing lymphatic vessels may hinder clearance of inflammatory cells and profibrotic molecules [24, 29]. Proteinuric kidney injury impairs contractility of renal collecting lymphatic vessels [24], which may be additive to the impairment caused by a high sodium environment. As in other tissues such as skin, immune cells promote leakiness of the expanded lymphatic vascular network, which further impairs interstitial clearance [65]. Whether sodium regulates lymphatic leakiness has yet to be determined. Immune cell: lymphatic interaction is further highlighted by findings that kidney injury activates a proinflammatory feedback loop involving the kidney lymph nodes driven by C-C chemokine ligand 21 (CCL21) stimulating recruitment of more CCR7 + DCs and lymphocytes [30]. Intrarenal inflammation and fibrosis could be attenuated by blocking the recruitment of CCR7^+^ cells or inhibiting lymphangiogenesis. Taken together, lymphangiogenesis in the setting of kidney injury and sodium may be context dependent such that in the early stages of injury, lymphangiogenesis serves to clear the interstitium and thus lessen the immune and fibrotic response. In contrast, lymphatic expansion accompanied by compromised absorptive capacity and/or leakiness of the collecting lymphatic vessels and dysfunctional lymphatic pumping dynamics will promote intrarenal stagnation of harmful molecules and cells that enhance kidney damage.

As discussed, sodium stimulates the APC formation of IsoLG, which is a powerful constrictor of renal collecting vessels [58]. Our new studies show that nephrotoxin injured proteinuric mice have an increased abundance of IsoLG-adduct containing monocytes and DCs compared to normal uninjured kidneys. Complementary studies of immune cell: LEC co-cultures found that a high salt environment stimulates the production of IsoLGs in APC and LECs. The relevance of these observations is underscored by findings that antagonism of IsoLG lessens immune cell activation and hypertension and reduces urinary IsoLG excretion together with improved kidney morphology and albuminuria [58, 66].

## Summary and Future Directions

Taken together, accumulating evidence indicates that sodium has direct cellular actions that are volume independent. Nonetheless, the prevailing clinical approach to mitigate adverse consequences of excess sodium is to limit its dietary intake and increase its excretion by targeting sodium transporters expressed along the tubular epithelium. We suggest that kidney disease-driven accumulation of sodium modulates non-tubular elements of the kidney parenchyma, namely, lymphatic endothelial cells and infiltrating immune cells. The high sodium environment following kidney injury stimulates potentially beneficial renal lymphangiogenesis but, in combination with increased intrarenal IsoLG, blunts lymphatic vessel function through pathways involving NKCC1 (Fig. 1). Future studies are needed to identify salt-sensing mechanisms that mediate harmful interactions between sodium-activated APCs and LECs in order to identify new targets for therapeutic interventions and prevention of progressive kidney injury.

## Key References

- Liu J, Shelton EL, Crescenzi R, Colvin DC, Kirabo A, Zhong J et al. Kidney Injury Causes Accumulation of Renal Sodium That Modulates Renal Lymphatic Dynamics. Int J Mol Sci. 2022;23(3). doi:10.3390/ijms23031428. This study demonstrates proteinurinc kidney disease promotes sodium accumulation in the renal interstitium and that a high sodium environment can diminish lymphatic vessel function via NKCC1 signaling.
- Zhong J, Yang HC, Shelton EL, Matsusaka T, Clark AJ, Yermalitsky V et al. Dicarbonyl-modified lipoproteins contribute to proteinuric kidney injury. JCI Insight. 2022;7(21). doi:10.1172/jci.insight.161878. This study demonstrated IsoLG-modified apoAI is increased in patients and animals with kidney disease and that IsoLG-apoAI contributes to renal disease progression by upregulating inflammatory cytokines, increasing oxidative stress in lymphatic endothelial cells, and compromizing lymphatic vessel pumping dynamics.
- Saleem M, Aden LA, Mutchler AP, Basu C, Ertuglu LA, Sheng Q et al. Myeloid-Specific JAK2 Contributes to Inflammation and Salt Sensitivity of Blood Pressure. Circ Res. 2024. doi:10.1161/circresaha.124.32359. This study identifies a mechanism by which sodium activates antigen presenting cell signaling pathways leading to IsoLG formation and T cell-mediated proinflammatory cytokine production in the context of salt sensitive blood pressure.

## Data Availability

No datasets were generated or analysed during the current study.
